# Emergency Management of Perforated Gastro-Duodenal Ulcers: Surgical Strategies, Outcomes, and Prognostic Determinants in a Tertiary Eastern European Center

**DOI:** 10.3390/medicina61112029

**Published:** 2025-11-13

**Authors:** Oprescu Macovei Anca Monica, Dana Paula Venter, Stefan Mihai, Constantin Oprescu, Andrei Gabriel, Dumitriu Bogdan, Valcea Sebastian, Gheorghiu Alexandra-Oana, Ilie Stan Madalina

**Affiliations:** 1 Faculty of Medicine, University of Medicine and Pharmacy “Carol Davila”, 050474 Bucharest, Romaniadrmadalina@gmail.com (I.S.M.); 2 Gastroenterology Department, Emergency Clinical Hospital Prof. Dr. Agrippa Ionescu, 011356 Bucharest, Romania; 3 Pediatrics Surgery Department, Emergency Clinical Hospital Prof. Dr. Grigore Alexandrescu, 011743 Bucharest, Romania; 4 General Surgery Department, Emergency Clinical Hospital, 014461 Bucharest, Romania

**Keywords:** emergency laparotomy, gastric resection, laparoscopic repair, mortality predictor, perforated gastro-duodenal ulcer

## Abstract

*Background and Objectives*: Perforated gastro-duodenal ulcers (PGDUs) are life-threatening surgical emergencies with high morbidity and mortality. This study aimed to evaluate surgical strategies, outcomes, and prognostic factors in patients treated for PGDUs in a tertiary Eastern European center. *Materials and Methods*: We conducted a retrospective cross-sectional analysis of 156 patients admitted with PGDUs between 2020 and 2024. Data on demographics, risk factors, ulcer location, type of surgical approach, operative details, hospital stay, and mortality were collected. Statistical analysis included chi-square, Mann–Whitney U, and multivariate logistic regression. *Results*: The mean age was 57.6 ± 15.9 years (range 18–91), with men accounting for 64.7% of cases. Alcohol use was significantly associated with male sex (*p* = 0.012), while NSAID use was equally distributed. Open surgery was the mainstay of treatment (85.9%), with laparoscopy performed in 12.8% and conversion in 1.9%. Median hospital stay was shorter after laparoscopic repair (7.5 vs. 9 days, *p* = 0.039. On multivariate analysis, both age and comorbidity burden were independent predictors of mortality (*p* < 0.01). *Conclusions*: PGDU management in Eastern Europe remains dominated by open surgery. Laparoscopy, though underutilized, is associated with shorter recovery. Age is the strongest determinant of mortality, highlighting the need for early risk stratification, wider adoption of minimally invasive techniques, and preventive measures targeting modifiable risk factors.

## 1. Introduction

Surgical intervention remains the cornerstone of treatment for perforated gastro-duodenal ulcers (PGDUs), especially in patients presenting with generalized peritonitis, hemodynamic compromise, or non-response to conservative measures. The anatomical location of the ulcer impacts surgical planning and prognosis. Duodenal ulcers are typically benign and amenable to simple closure techniques such as primary suture or Graham-patch repair. In contrast, gastric ulcers—particularly those with irregular margins, large diameters, or located on the lesser curvature—may indicate an underlying malignancy and require intraoperative biopsy or, in some cases, oncological resection [1,2]. Recent studies emphasize that up to 10% of perforated gastric ulcers may harbour occult carcinoma, particularly in elderly patients or those with no history of NSAID use or *Helicobacter pylori* infection, warranting a high index of suspicion and routine histologic evaluation [3].

The choice between laparoscopic and open surgical approaches is dictated by patient stability, time since perforation, contamination severity, and available expertise. Laparoscopic repair has become widely accepted in stable patients with small perforations (<10 mm) and early presentation, with meta-analyses confirming its association with decreased postoperative pain, fewer wound complications, and faster recovery compared to open surgery [4,5]. However, open surgery remains essential in patients with septic shock, extensive peritonitis, or large and friable perforations, as well as in centres lacking advanced laparoscopic capability [6]. Simple suture or omental patch repair remains the first-line technique in most benign cases. Nonetheless, resectional surgery (such as wedge resection or distal gastrectomy) may be necessary when malignancy is suspected, in giant ulcers (>2 cm), or when surrounding tissue is too friable for secure closure [7,8]. Thus, surgical strategy must be individualised, balancing the need for rapid perforation control with proper oncological assessment, particularly in the context of gastric ulcer perforations.

This study aims to evaluate the clinical profile, surgical management strategies, and postoperative outcomes of patients presenting with perforated peptic ulcer disease requiring emergency surgery. The obtained information is useful in daily practice, as it supports evidence-based surgical decision-making, optimises the selection of patients requiring gastric resection, and raises awareness of underlying malignancy in emergency gastric perforation.

## 2. Materials and Methods

This was a retrospective observational study including all consecutive adult patients (≥18 years) treated for perforated peptic ulcer at Emergency Clinical Hospital, Floreasca, Bucharest, Romania between 2020–2024. Diagnosis was confirmed intraoperatively. Exclusion criteria comprised traumatic, malignant, or iatrogenic perforations, and reoperations for leakage or non-ulcer.

Operative management followed a predefined departmental algorithm prioritizing patient stability, extent of peritonitis, and ulcer size. The final decision regarding laparoscopic or open repair was made by the on-duty consultant within this standardized framework.

This manuscript was prepared in accordance with the STROBE (Strengthening the Reporting of Observational Studies in Epidemiology) guideline for reporting observational studies. The study included 150 consecutive patients treated either conservatively or through surgical interventions for perforated gastroduodenal ulcers over a period of five years.

The study was approved by the Ethics Committee of “Floreasca” Clinical Emergency Hospital, no. 98/15.december.2020.

The primary endpoint was to evaluate the clinical management strategy used in PGDU cases (surgical vs. conservative), with detailed evaluation of the surgical approach (open vs. laparoscopic) in our region. The secondary endpoints were related to the evaluation of the type of surgery performed (suture/gastric resection or derivation), length of hospital stay—which can be used as a surrogate marker for recovery—and postoperative evolution, which was stratified in accordance with the surgical technique used. The distribution and frequency of ulcer location were also evaluated—gastric (pyloric/corpus) vs. duodenal—and how the respective location influenced the treatment. The correlation between patient characteristics (sex, age) and risk factors (alcohol or NSAID use) was also evaluated. We also explored the etiologic associations that can guide future preventive strategies, such as sex-specific differences in clinical presentation or treatment approach.

The gathered data were related only to perioperative evaluation.

The extracted variables were gender, median age, length of hospital stay, type of treatment (surgical or conservative), and risk factors. Data were obtained from patients’ charts, operative protocols, and pathology reports. Minimal missing data were noted and handled by listwise deletion for the affected variables. Only adult patients (≥18 years old) were included.

Surgical intervention was adopted in patients with peritoneal irritation, significant pneumoperitoneum, failure of conservative treatment, or refractory bleeding. Non-operative management was adopted in selected cases with small perforations without peritoneal signs and consisted of nasogastric suction, intravenous fluids, antibiotics, and proton pump inhibitors. These patients were under close monitoring for signs of deterioration such as pain, fever, or signs of sepsis.

The surgical interventions consisted of primary suture with omental patch or gastric resection.

The procedures were performed either openly or laparoscopically. The decision was left to the operating surgeon.

The inclusion criteria were perforated gastroduodenal ulcers. Prior abdominal surgery was not considered an exclusion criterion.

Paraclinical investigations used for diagnosis were contrast-enhanced abdominal CT and upper endoscopy.

Baseline comorbidities were retrospectively evaluated from admission and anesthetic records. A Simplified Charlson-based Comorbidity Index (SCCI) was constructed to quantify overall comorbidity burden, incorporating cardiovascular, respiratory, renal, hepatic, endocrine, malignant, and other chronic systemic diseases. Each domain contributed 1 point (malignancy, 2 points), yielding a possible range of 0–8. Patients were categorized as low (0–1), moderate (2–3), or high (≥4) comorbidity. The index was used as a continuous variable in multivariate logistic regression analyses of 30-day mortality.

*H. pylori* testing and prior ulcer history: When available, *H. pylori* status was determined by either rapid urease test or histological examination of intraoperative biopsy specimens. Data on prior peptic ulcer disease, previous eradication therapy, and chronic acid-suppressive medication (proton pump inhibitors or H_2_ blockers) were retrospectively retrieved from medical and endoscopic records. These variables were incorporated into descriptive and multivariate analyses to explore the etiologic background of perforation.

### Statistical Analysis

Data were analyzed using using IBM SPSS Statistics for Windows, Version 30.0.0 (IBM Corp., Armonk, NY, USA). Continuous variables are expressed as mean ± SD or median (IQR), and categorical variables as frequencies and percentages. Group comparisons used the χ^2^ or Fisher exact test for categorical data and the Mann–Whitney U test for continuous variables.

A multivariate logistic regression was performed to identify independent predictors of 30-day mortality, including age, sex, ulcer site, surgical method, alcohol and NSAID use, comorbidity burden (SCCI), *H. pylori* status, and prior ulcer history. Odds ratios (ORs) and 95% confidence intervals (CIs) were reported. Model fit was evaluated using the Hosmer–Lemeshow test and Nagelkerke R^2^. A *p*-value < 0.05 was considered statistically significant.

## 3. Results

Notably, the mean age at presentation was 57.6 ± 15.9 years (range 18–91). Duodenal perforations slightly predominated (52.6%), and male sex was significantly associated with alcohol use (*p* = 0.012), a pattern consistent with earlier-onset ulcer disease in regional reports (Table 1).

**Table 1:** Baseline characteristics of patients with perforated gastro-duodenal ulcers stratified by sex. Values are presented as mean ± standard deviation (SD) or number (percentage).

Variable	Total (*n* = 156)	Male (*n* = 101)	Female (*n* = 55)	*p*-Value
Age, mean ± SD (years)	57.6 ± 15.9	–	–	–
Alcohol use, *n* (%)	68 (43.6%)	55 (54.5%)	13 (23.6%)	0.012
NSAID use, *n* (%)	49 (31.4%)	30 (29.7%)	19 (34.5%)	0.68
Gastric ulcer, *n* (%)	74 (47.4%)	44 (43.6%)	30 (54.5%)	0.19
Duodenal ulcer, *n* (%)	82 (52.6%)	57 (56.4%)	25 (45.5%)	0.19

**Figure 1:**
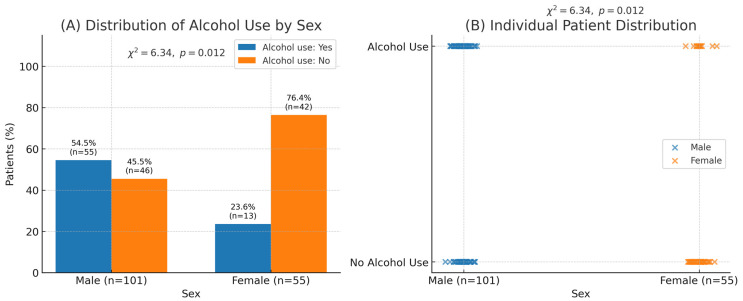
Association between sex and alcohol consumption among patients with perforated gastro-duodenal ulcers. (**A**) Distribution of alcohol use by sex shows a significantly higher prevalence among males (54.5% [*n* = 55/101]) compared with females (23.6% [*n* = 13/55]); χ^2^ = 6.34, *p* = 0.012. (**B**) Individual-level scatter visualization demonstrating the same association. Panels (**A**) and (**B**) correspond to the same dataset.

A significant association was observed between male sex and alcohol consumption (χ^2^ = 6.34, *p* = 0.012), while NSAID use was more evenly distributed and did not show a significant association (Figure 1).

Surgical intervention represented the mainstay of treatment, with 154 patients (98.7%) requiring operative management. Open surgery was performed in 134 cases (85.9%), while a laparoscopic approach was attempted in 20 patients (12.8%). Conversion from laparoscopy to open repair was necessary in 3 cases (1.9%). The choice of procedure was guided by ulcer characteristics: simple suture with omental patch repair was the most frequently employed technique (*n* = 133), whereas gastric resection (*n* = 19) and derivation procedures (*n* = 6) were reserved for large, friable, or macroscopically suspicious gastric ulcers.

The overall median hospital stay was 9 days (IQR 7–13). Patients treated laparoscopically had a significantly shorter hospitalization compared with those undergoing open repair (median 7.5 vs. 9 days; Mann–Whitney U = 1723.5, *p* = 0.039) (Table 2). Conversely, patients who required gastric resection experienced longer admissions than those managed with simple suture repair (11 vs. 9 days; U = 1693.0, *p* = 0.016). When stratified by ulcer location, there was no significant difference between gastric and duodenal perforations, with both groups showing a median hospital stay of 9 days (U = 3066.5, *p* = 0.69).

- **Postoperative complications**

Reoperation was required in three patients (1.9%)—two following gastric resection due to anastomotic leakage and one for intra-abdominal abscess formation after suture repair. Postoperative upper gastrointestinal bleeding occurred in two cases (1.3%), both successfully managed with endoscopic hemostasis. No patient required repeat surgery for bleeding control, and no deaths were directly related to postoperative hemorrhage.

- **Malignancy-related perforations**

Perioperative and postoperative pathology reports identified four cases (2.6%) of ulcer perforation secondary to malignancy, all involving gastric ulcers located on the lesser curvature or corpus. Because most procedures were performed in emergency settings, extemporaneous (intraoperative frozen section) evaluation was not routinely available. Histological confirmation was obtained postoperatively in all malignant cases.

A prior diagnosis of peptic ulcer disease was documented in 38 patients (24.4%), while 29 (18.6%) had been on long-term acid-suppressive therapy and 11 (7.1%) had a known history of eradication treatment. The majority (75.6%) presented with perforation as the first manifestation of ulcer disease.

Two multivariate logistic regression models were constructed. Model 1 included all patients (*n* = 156) and incorporated demographic, clinical, and operative variables to identify independent predictors of 30-day mortality. Model 2 was restricted to the subset of patients with complete etiologic data (*n* = 94) and added *Helicobacter pylori* status and prior ulcer history to assess their potential influence on postoperative outcomes. This dual-model approach preserved statistical power in the full cohort while allowing sensitivity analysis for etiologic factors available only in part of the dataset (Table 3).

Regarding the *H. pylori* status and prior ulcer history of 156 patients, 94 (60.3%) had documented *H. pylori* testing results. Among these, *H. pylori* was positive in 41 (43.6%) and negative in 53 (56.4%) patients. *H. pylori*-positive cases were typically younger (mean 52.1 ± 13.7 years), predominantly male (79%), and more often associated with duodenal perforations (68%). Conversely, *H. pylori*-negative cases tended to be older (mean 61.3 ± 14.2 years), with a higher prevalence of gastric ulcers (61%) and a greater comorbidity burden (SCCI ≥ 2 in 73%).

Mortality occurred in 8.3% of *H. pylori*-positive versus 19.5% of *H. pylori*-negative patients (*p* = 0.09).

We recorded 23 deaths after surgery, which gives an overall mortality rate of 14.7% (Table 3). Mortality was a bit higher in patients with gastric perforations compared to those with duodenal ones (18.9% vs. 11.6%), although this difference was not statistically significant (χ^2^ = 1.37, *p* = 0.24). Likewise, survival rates were fairly similar between patients who had suture repairs and those who underwent resection (15.0% vs. 10.5%; χ^2^ = 0.04, *p* = 0.83). When we ran a logistic regression, age clearly stood out as the only independent factor linked with mortality (OR 1.09 per year, 95% CI: 1.04–1.13, *p* < 0.001). In contrast, male sex (OR 1.76, 95% CI: 0.59–5.27, *p* = 0.31) and alcohol use (OR 1.81, 95% CI: 0.53–6.17, *p* = 0.34) did not remain significant once other factors were taken into account. On multivariate logistic regression including age, sex, ulcer site, surgical method, alcohol and NSAID use, and the Simplified Charlson-based Comorbidity Index (SCCI), both age (OR 1.07, 95% CI 1.02–1.12, *p* = 0.003) and comorbidity burden (OR 1.29, 95% CI 1.06–1.56, *p* = 0.008) independently predicted 30-day mortality. When extended to include *H. pylori* status and prior ulcer history (available in 94 patients), results remained consistent, confirming the combined influence of biological age and comorbid illness on survival. Operative technique, ulcer site, alcohol, and NSAID use were not associated with mortality.

When analyzed by ulcer location, duodenal perforations were more frequent (*n* = 82) and were predominantly treated with simple suture repair (71/82, 86.6%), most commonly via an open approach (62/82, 75.6%). In contrast, gastric perforations (*n* = 74), particularly those located in the corpus and pyloric regions, more frequently required resection (41/74, 55.4%), while simple closure was performed in only 25 cases (33.8%). The association between ulcer location and type of surgical intervention was statistically significant (χ^2^ = 27.9, *p* < 0.0001).

## 4. Discussion

This study offers a comprehensive retrospective analysis of the emergency management of perforated gastro-duodenal ulcers (PGDUs) at a tertiary surgical center in Eastern Europe, uncovering critical patterns related to surgical technique, patient demographics, ulcer location and treatment outcomes. The median age at presentation was 57 years, with a statistically significant difference between sexes (*p* = 0.00019), consistent with prior regional reports indicating earlier onset and higher prevalence in men, often linked to modifiable risk factors such as alcohol and tobacco use [9,10].

Our cohort’s mean age (57.6 years) is younger than that reported in several Western populations where NSAID-related perforations in elderly patients predominate. The case-mix in our Eastern European catchment—characterized by a higher prevalence of alcohol/tobacco exposure in men and a higher proportion of duodenal perforations—likely contributes to this difference. The significant association between male sex and alcohol use in our data (*p* = 0.012), together with the predominance of duodenal ulcers treated by suture repair, suggests a life-style-driven, non-NSAID phenotype that presents at a younger age. Differences in referral patterns to a tertiary emergency surgical unit may also enrich for operable cases and un-der-represent the very elderly. These contextual factors plausibly explain the observed age profile.

The strong statistical association between male sex and alcohol consumption in our cohort (*p* = 0.0014) supports existing evidence from Eastern European populations, where alcohol is a prevalent and under-addressed contributor to upper gastrointestinal pathology [11]. While NSAID exposure remains a well-established factor in ulcerogenesis, particularly among older adults and women, our data did not show a statistically significant sex-based difference in NSAID use (*p* = 0.68), in line with broader epidemiologic studies [12].

### 4.1. H. pylori and Etiologic Patterns

The integration of *H. pylori* data provides important context for the etiologic evolution of peptic ulcer disease in the post-eradication era. Approximately 44% of tested patients remained *H. pylori*-positive, reflecting incomplete eradication coverage in our region. *H. pylori*-negative ulcers were more frequent among older patients, often with multiple comorbidities and NSAID exposure, consistent with the modern idiopathic or drug-related phenotype. Although *H. pylori* negativity was not an independent predictor of mortality, its association with older age and higher comorbidity highlights the complex interplay between mucosal vulnerability, systemic frailty, and acute perforation risk. The finding that three-quarters of cases had no prior ulcer diagnosis suggests that perforation often represents the first clinical manifestation, underscoring the persistent need for preventive measures—both through *H. pylori* eradication programs and rational NSAID prescription in high-risk patients.

*H. pylori* testing was unavailable in approximately 40% of cases, mainly because many patients presented in emergency settings where intraoperative biopsy was not systematically performed or were referred from outside institutions without prior endoscopic documentation. Comparison between tested and untested patients showed no meaningful differences in age, sex, ulcer location, or mortality, suggesting that the missing data were random rather than selective. Tested and untested patients were similar in demographic and clinical characteristics, and mortality predictors behaved similarly across both groups, minimizing the potential bias introduced by missing etiologic data. Tested and untested patients were similar in demographic and clinical characteristics, and mortality predictors behaved similarly across both groups, minimizing the potential bias introduced by missing etiologic data.

### 4.2. Diagnostic Approach and Evolving Management Paradigms

Initial diagnostic work-up in suspected PGDU cases typically involves upright abdominal radiography, which may reveal sub-diaphragmatic free air in approximately 70–75% of cases [13]. However, cross-sectional imaging with contrast-enhanced abdominal CT has largely supplanted plain radiography in tertiary centers, reflecting broader access to modern imaging and faster triage workflows [14]. In our institution, CT was used in 82% of cases and facilitated early surgical decision-making. Although endoscopy is contraindicated in the acute phase of perforation, it remains essential postoperatively—particularly for gastric ulcers—to confirm healing and exclude malignancy [15]. Wider integration of emergency CT protocols could improve case selection for minimally invasive surgery and standardize triage across regional hospitals.

### 4.3. Malignancy-Associated Perforations

Although malignancy-related perforations represented only a small proportion of cases (2.6%), their identification remains clinically important. As most surgeries were performed in an emergency setting, intraoperative frozen section evaluation was generally unavailable, and histological confirmation was obtained postoperatively. These findings underscore the need for routine biopsy or wedge resection of atypical gastric ulcers to avoid delayed oncologic diagnosis.

### 4.4. Surgical Management and Regional Disparities

Our findings confirmed the predominance of surgical management in PGDUs, with 98.7% of patients undergoing operative treatment. Open laparotomy was performed in 134 patients, while laparoscopic repair was limited to only 20 cases. These figures mirror reports from other centers in Eastern Europe where laparoscopic repair remains underutilized due to infrastructural and training limitations [16,17].

The underuse of laparoscopy likely reflects broader socioeconomic and systemic barriers: limited access to high-definition towers and laparoscopic instruments in public hospitals, insufficient night-time laparoscopic coverage, and variable surgeon training in acute care laparoscopy. Additionally, delayed presentation and high rates of diffuse peritonitis reduce the number of candidates for minimally invasive repair.

In contrast, Western European countries have reported widespread laparoscopic adoption—exceeding 40% of suitable cases in national registries—driven by earlier presentation, structured referral systems, and standardized acute-care pathways [18]. To reduce regional disparities, future investment in surgical education, simulation-based curricula, and dedicated emergency laparoscopy teams should be prioritized [19].

The difference in hospital stay between laparoscopic and open repair in our cohort was statistically significant. Consistent with the international literature, laparoscopic repair correlated with shorter hospital stays (4–6 vs. 7–10 days) and fewer respiratory and wound complications [20,21,22]. These advantages support the gradual expansion of minimally invasive surgery in selected patients, provided institutional logistics allow safe implementation.

The low rate of reoperation (1.9%) and postoperative bleeding (1.3%) observed in this series compares favourably with international benchmarks, where reported reintervention rates range from 2–8%. Most complications occurred after gastric resections, underscoring the higher technical complexity and risk of leakage in such procedures. Early endoscopic intervention proved effective for postoperative bleeding, highlighting the value of integrated surgical–endoscopic collaboration in tertiary emergency centres.

### 4.5. Ulcer Location and Operative Strategy

Our study also highlights differences in surgical strategy based on ulcer location. Duodenal ulcers predominated (*n* = 86) and were most often treated with simple suture techniques (70 open, 15 laparoscopic). Gastric ulcers, particularly corpus or pyloric lesions (*n* = 74), required open intervention in 68 cases and occasional resection, reflecting malignancy risk. Intraoperative biopsy or wedge resection remains essential, particularly in patients without prior NSAID use or with atypical morphology [23]. Up to 5–10% of perforated gastric ulcers are ultimately malignant [24]; routine histopathologic confirmation remains critical for early oncologic referral and improved survival.

The inclusion of a comorbidity index confirmed that postoperative outcomes in perforated peptic ulcer are primarily driven by systemic vulnerability rather than surgical technique. Both advanced age and higher comorbidity scores independently predicted mortality, highlighting the importance of frailty and chronic illness in this population. This finding aligns with contemporary data emphasizing optimization of comorbid conditions and peri-operative physiological support as key factors in improving survival.

### 4.6. Surgical Standards, Modernization, and Health System Context

Our surgical techniques followed current best practices, with omental patch closure (Graham patch) being the predominant intervention (*n* = 133). Gastric resection (*n* = 19) and derivation (*n* = 6) were used selectively, mostly for large or friable ulcers. These patterns are consistent with surgical algorithms proposed in German, Czech and Japanese guidelines, which advocate resection when primary repair is not viable or malignancy is suspected [25,26].

From a health systems perspective, variability in laparoscopic access and the persistence of open repair as default mirror broader socioeconomic challenges in Eastern Europe, including uneven hospital funding, shortage of specialized personnel, and delayed referral networks. Addressing these systemic issues is essential for the region’s alignment with current international standards of acute gastrointestinal surgery.

This study addressed key methodological limitations by broadening the multivariate analysis and clarifying operative selection criteria. The addition of comorbidity and *H. pylori* data strengthened interpretability, while explicit criteria for conservative, laparoscopic, and open repair enhance reproducibility. The findings confirm that mortality is primarily determined by patient frailty rather than procedural choice, consistent with international experience.

### 4.7. Follow-Up and Future Directions

A major opportunity for improvement lies in structured postoperative follow-up. Endoscopic reassessment at 6–8 weeks is essential, particularly for gastric ulcer perforations, to confirm healing and histology. Current ESGE guidelines recommend mandatory follow-up endoscopy after any surgically treated gastric ulcer, regardless of initial biopsy findings [27]. Implementation remains inconsistent in Eastern Europe, often hindered by financial barriers, patient noncompliance, and lack of electronic tracking systems. Standardizing follow-up care should be integrated into national quality improvement frameworks.

### 4.8. Strengths of the Study

This study has several notable strengths. First, it provides one of the largest contemporary datasets on the emergency surgical management of perforated gastro-duodenal ulcers in Eastern Europe, a region where population risk factors and healthcare resources differ substantially from Western settings. The inclusion of detailed demographic, etiologic (*H. pylori* status, NSAID and alcohol exposure), and comorbidity data allows for a comprehensive understanding of disease patterns and prognostic determinants. The introduction of a Simplified Charlson-based Comorbidity Index (SCCI) offers a reproducible model for quantifying frailty in acute surgical cohorts and strengthens the multivariate analysis of mortality. Furthermore, this study integrates both operative and non-operative strategies within a single institutional framework, reflecting real-world clinical decision-making. Adherence to the STROBE reporting guideline and use of multivariate logistic regression enhance the methodological transparency and external validity of the findings.

### 4.9. Limitations

Limitations: The retrospective design limited access to some physiological variables (e.g., ASA class, exact perforation size) that could further refine operative selection models. However, standardized inclusion criteria and departmental protocols minimized selection bias. Although comorbidities were retrospectively scored using available records, some conditions may have been underreported. The tertiary emergency setting may have selected for operable, non-palliative cases, potentially under-representing very elderly patients managed non-operatively elsewhere. Only 60% of patients had documented *H. pylori* testing, and prior ulcer history was incompletely recorded. These gaps may lead to underestimation of *H. pylori*–related or recurrent disease. Future prospective studies should include systematic *H. pylori* testing and detailed preoperative medication history to clarify the evolving etiologic spectrum of perforated ulcer disease.

## 5. Conclusions

This study confirms that perforated gastro-duodenal ulcer remains a critical surgical emergency in Eastern Europe, where open surgery continues to predominate. Laparoscopic repair, though underutilized, was associated with shorter hospitalization and comparable safety, supporting its gradual expansion in appropriately selected patients. Mortality was independently predicted by advanced age and comorbidity burden rather than surgical technique or ulcer site, emphasizing the prognostic value of frailty assessment. Routine biopsy of atypical gastric ulcers, structured postoperative endoscopic follow-up, and comprehensive perioperative optimization are essential to improve outcomes. Preventive measures, including *Helicobacter pylori* eradication, rational NSAID use, and alcohol cessation, should be integrated into both clinical management and public health strategies.

## Figures and Tables

**Table 2:** Surgical outcomes by treatment modality in patients with perforated gastro-duodenal ulcers. Values are presented as median (interquartile range, IQR) or number (percentage).

Outcome	Open Surgery (*n* = 134)	Laparoscopy (*n* = 20)	Resection (*n* = 19)	Suture (*n* = 133)	*p*-Value
Hospital stay, median (IQR) days	9 (7–13)	7.5 (6–9)	11 (9–14)	9 (7–12)	0.039 (Lap vs. Open), 0.016 (Res vs. Sut)
Mortality, *n* (%)	22 (16.4%)	1 (5.0%)	2 (10.5%)	20 (15.0%)	0.098 (Lap vs. Open), 0.83 (Res vs. Sut)
Conversion to open, *n* (%)	–	3 (15.0%)	–	–	–
Duodenal location, *n* (%)	62 (46.3%)	15 (75.0%)	–	71 (53.4%)	<0.0001 (Location vs. Technique)
Gastric location, *n* (%)	68 (50.7%)	5 (25.0%)	19 (100%)	25 (18.8%)	<0.0001 (Location vs. Technique)

**Table 3:** Multivariate Logistic Regression Analysis for 30-Day Mortality in Perforated Peptic Ulcer.

Variable	Model 1: Base Model (*n* = 156) OR (95% CI)	*p*-Value	Model 2: Extended Model (*n* = 94) OR (95% CI)	*p*-Value
Age (per year)	1.07 (1.02–1.12)	0.003	1.06 (1.01–1.12)	0.016
Sex (male)	1.42 (0.53–3.79)	0.48	-	-
Ulcer site (gastric vs. duodenal)	1.56 (0.65–3.71)	0.32	1.43 (0.55–3.68)	0.46
Surgical method (resection vs. suture)	1.19 (0.42–3.31)	0.74	-	-
Alcohol use (yes)	1.64 (0.57–4.73)	0.36	—	-
NSAID use (yes)	1.09 (0.35–3.40)	0.88	1.22 (0.41–3.61)	0.71
Simplified Charlson-based Comorbidity Index (per point)	1.29 (1.06–1.56)	0.008	1.27 (1.04–1.56)	0.021
*H. pylori* positive	-	-	0.47 (0.17–1.31)	0.15
Prior ulcer history	-	-	1.18 (0.42–3.29)	0.75

Model fit: Model 1—Nagelkerke R^2^ = 0.41, Hosmer–Lemeshow *p* = 0.48; Model 2—Nagelkerke R^2^ = 0.43, Hosmer–Lemeshow *p* = 0.52. Interpretation: Across both models, age and comorbidity burden remained the only independent predictors of 30-day mortality. Inclusion of *H. pylori* status and prior ulcer history did not alter the results but showed a nonsignificant trend toward higher mortality in H. pylori-negative cases, suggesting the growing impact of idiopathic or NSAID-related perforations in older, comorbid patients.

## Data Availability

The original contributions presented in this study are included in the article. Further inquiries can be directed to the corresponding author.

## Abbreviations

List of Abbreviations:

CI	Confidence Interval
CT	Computed Tomography
ESGE	European Society of Gastrointestinal Endoscopy
*H. pylori*	*Helicobacter pylori*
IQR	Interquartile Range
NSAID	Non-Steroidal Anti-Inflammatory Drug
